# Inorganic Bottlebrush and Comb Polymers as a Platform for Supersoft, Solvent-Free Elastomers

**DOI:** 10.1021/acspolymersau.3c00043

**Published:** 2024-01-11

**Authors:** Edip Ajvazi, Felix Bauer, Paul Strasser, Oliver Brüggemann, Rene Preuer, Milan Kracalik, Sabine Hild, Mahdi Abbasi, Ingrid Graz, Ian Teasdale

**Affiliations:** † Institute of Polymer Chemistry, Johannes Kepler University Linz, Altenberger Straße 69, 4040 Linz, Austria; ‡ Christian Doppler Laboratory for Soft Structures for Vibration Isolation and Impact Protection (ADAPT), School of Education, STEM Education, Johannes Kepler University Linz, Altenberger Straße 69, 4040 Linz, Austria; § Institute of Polymer Science, Johannes Kepler University Linz, Altenberger Straße 69, 4040 Linz, Austria; ∥ Borealis Polyolefine GmbH, Innovation Headquarters, St.-Peter-Straße 25, 4021 Linz, Austria

**Keywords:** bottlebrush polymers, supersoft elastomers, polyphosphazenes, polydimethylsiloxane, inorganic polymers

## Abstract

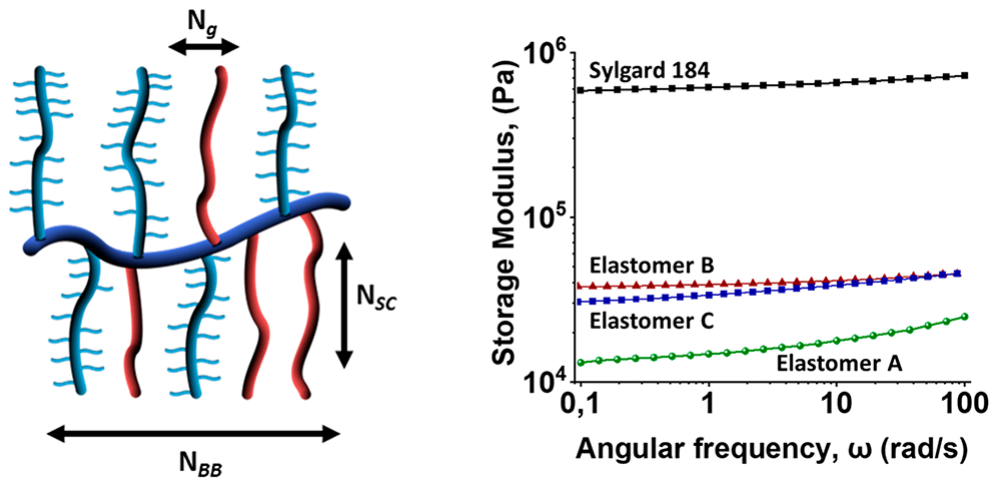

Due to their unique rheological and mechanical properties, bottlebrush polymers are inimitable components of biological and synthetic systems such as cartilage and ultrasoft elastomers. However, while their rheological properties can be precisely controlled through their macromolecular structures, the current chemical spectrum available is limited to a handful of synthetic polymers with aliphatic carbon backbones. Herein we design and synthesize a series of inorganic bottlebrush polymers based on a unique combination of polydimethylsiloxane (PDMS) and polyphosphazene (PPz) chemistry. This non-carbon-based platform allows for simple variation of the significant architectural dimensions of bottlebrush-polymer-based elastomers. Grafting PDMS to PPz and vice versa also allows us to further exploit the unique properties of these polymers combined in a single material. These novel hybrid bottlebrush polymers were cured to give supersoft, solvent-free elastomers. We systematically studied the effect of architectural parameters and chemical functionality on their rheological properties. Besides forming supersoft elastomers, the energy dissipation characteristics of the elastomers were observed to be considerably higher than those for PDMS-based elastomers. Hence this work introduces a robust synthetic platform for solvent-free supersoft elastomers with potential applications as biomimetic damping materials.

## Introduction

Recently, elastomers from bottlebrush polymers have come to the fore in their ability to be mechanically soft, like gels, but at the same time firm, elastic, durable, and without dependence on volatiles.^1,2^ This development in synthetic chemistry has been followed by a drive toward soft materials that can truly mimic the unique mechanical performance of living tissues.^3,4^ Living tissues can perform a wide range of purposes within a single material, namely, being considerably softer than, for example, synthetic polymer materials yet firm due to their dynamic strain-stiffening behavior. They are also defined by relatively high strength. An additional feature of living soft tissue is a relatively high damping factor (tan δ) ranging from 0.1 of skin to 0.7 of brain tissue.^3^ This damping behavior protects tissues from damage, enabling them to dissipate energy in a broad frequency range.

In recent years Sheiko and Dobrynin have demonstrated that the advanced architectural features of bottlebrush polymers can not only be used to tune softness but also for strain-stiffening, strength, and damping.^5^ These findings open the door to a new domain, previously unattainable for linear network polymers, be it gels or solvent-free elastomers, moving toward synthetic soft-materials, which are concurrently firm and soft, elastic, and energy dissipating.^3^ Bottlebrush polymers are commonly defined as macromolecules grafted with a high density of polymer side-chains, giving them distinctive mechanical and rheological properties.^6^ On top of the fundamental chain-stiffness of the polymer chains, the physical properties of bottlebrush polymers are defined by three independently controllable architectural parameters, the length of the backbone (*N*_BB_), side chains (*N*_SC_), and the average degree of polymerization (DP) between two neighboring branching points, *N*_g_ = *M*_g_/*M*^0^, or the grafting density (*N*_g_^–1^).^7^ For cured elastomers, the distance between cross-links, *N*_x_, as well as the length of the cross-linker (*N*_CL_) can also be used to tune their fundamental properties,^1^ while dynamic bonding^8^ or phase separation^9,10^ may be used to introduce self-healing and processability. The extremely low chain entanglement in high-density bottlebrush polymers leads to supersoft materials having a rubber plateau modulus similar to that of gels (10^2^–10^5^ Pa).^5,7,11^ Furthermore, elastomers based on bottlebrush polymers are inherently soft and have a high damping factor due to their so-called “dangling ends”, which increase the number of relaxation modes, helping to dissipate energy.^12,13^

Although a number of molecular brushes have been synthesized and studied, the majority of these bottlebrush polymers have backbones that are based on carbon–carbon bonds, most predominantly poly(methacrylate)^14^ and polynorbornenes.^15^ However, exploring molecular brushes built using novel polymer backbones could open the way to novel material properties.^16^ In recent years, there has been an increasing interest in polymers that incorporate phosphorus and silicon, which have a diverse range of applications, in particular in biomedicine.^17^ While polysiloxanes are well-established, phosphorus-based polymers are less commercially developed but are especially appealing because phosphorus is a common element in the human body and plays a crucial role in many biochemical processes. Nature seems to prefer phosphorus because of its multivalency, which enables it to carry a negative charge and polymerize simultaneously, as well as its capacity to control binding through reversible hydrolysis.^18^ Phosphorus-based polymers, including polyphosphazenes, polyphosphoesters and polyphosphoramidates, are also of interest for their easily tunable biodegradation to non-cytotoxic and predictable degradation products.^19,20^ New, highly controlled synthesis routes have facilitated their use as tools for self-assembly, polymer therapeutics,^21^ vaccine delivery agents,^22^ tissue engineering,^23^ and biomedical coatings.^24^ Recently, we have prepared fully water-soluble bottlebrush polyphosphazenes (PPz) as nanomedicines with unique biodistribution profiles.^25^ A distinctive feature of PPz is low stiffness; in fact, polydifluorophosphazene has the lowest barrier to rotation ever calculated for skeletal bonding in polymers,^26^ significantly lower than C–C bonds. Furthermore, the polyphosphazene backbone allows unprecedentedly dense branching (*N*_g_^–1^ = 2). The high grafting density is possible due to the unique bonding situation with two easily functionalized groups per repeat unit that are both highly reactive and accessible due to their being on different sides of the chains of the backbone. Herein we exploit the high valency and molecular flexibility to prepare novel bottlebrush copolymers with PDMS, use them to prepare supersoft elastomers, and study their energy-dissipating properties.

## Results

### Bottlebrush PPz-PDMS

**Scheme 1:**
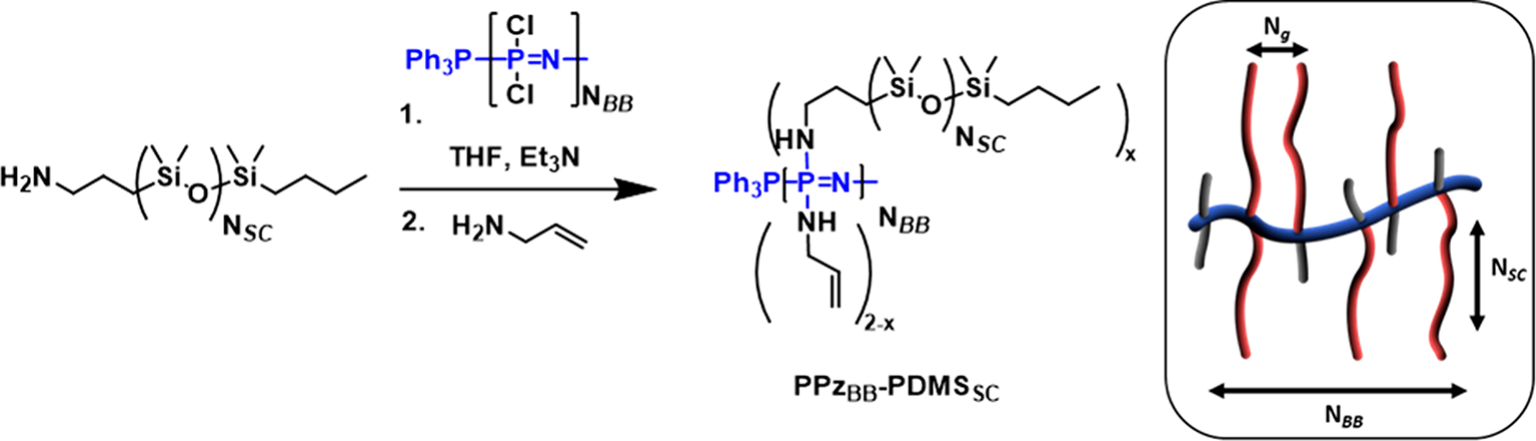
Synthesis of PDMS Grafted Polyphosphazenes with Allyl Functional Groups as Subsequent Curing Sites

**Figure 1:**
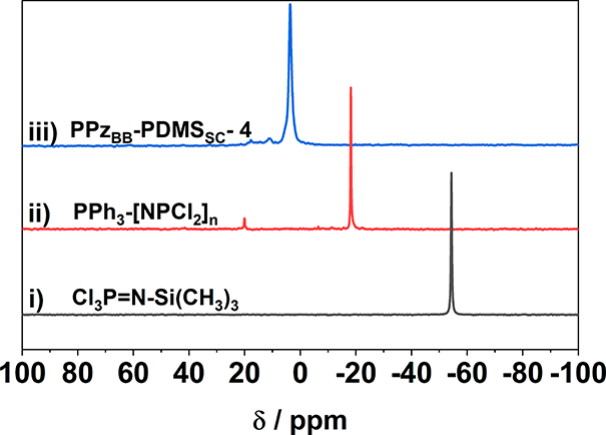
^31^P NMR spectra of the monomer trichlorophosphoranimine Cl_3_PNSiMe_3_ (i), polydichlorophosphazene [NPCl_2_]_*n*_, including the signal of the PPh_3_ end group, (ii) and the macrosubstituted PDMS grafted polyphosphazene **PPz**_**BB**_**-PDMS**_**SC**_**-4** (iii) demonstrating full conversion of [NPCl_2_]_*n*_ to the bottlebrush polymer (and thus *N*_g_ = 0.5).

The inorganic backbone [NPCl_2_]_*n*_ was first prepared from a phosphine-mediated polymerization according to literature procedures.^27^ The chain length, and thus ultimate *N*_BB_ of the resulting bottlebrush polymers, is determined by the ratio of PPh_3_Cl_2_ initiator and can be estimated by ^1^H NMR spectroscopy (Figure SI-1). The uniquely high conformational flexibility of the [NPCl_2_]_*n*_ backbone, in combination with the excellent leaving group ability of the chloride atoms, makes grafting-to a viable approach to bottlebrush **PPz**_**BB**_**-PDMS**_**SC**_ polymers. To achieve this, PDMS-NH_2_ was grafted to [NPCl_2_]_*n*_ in different amounts (*x* = 0.2, 0.6, and 1.8 equiv) to give **PPz**_**BB**_**-PDMS**_**SC**_**-1–4** (Scheme 1 and Table 1). An excess of allylamine was added to provide curing sites for subsequent elastomers and ensured a complete substitution of the chloride atoms. The excess allyl amine could be easily removed by evaporation, and the triethylamine salts were washed from the hydrophobic polymers with H_2_O. The facile postpolymerization substitution was analyzed by ^31^P NMR spectroscopy (Figure 1), confirming the complete substitution of [NPCl_2_]_*n*_ to [NPR_2_]_*n*_. To also explore the upper limit for the grafting of PDMS to [NPCl_2_]_*n*_ experiments were conducted where PDMS-NH_2_ (*N*_SC_ = 26) was used as the sole substituent. Here it was observed that indeed complete saturation (*x* = 2) of the PPz backbone with PDMS side chains was possible (Figure SI-2). To confirm the steric accessibility of the allyl groups as curing sites in **PPz**_**BB**_**-PDMS**_**SC**_, we conducted a further experiment in which an excess of monohydride PDMS was added to **PPz**_**BB**_**-PDMS**_**SC**_**-1**. ^1^H NMR spectroscopy showed a quantitative conversion of the allyl groups (Figure SI-3) and thus confirmed their availability for further reactions.

**Table 1:** Structural Characteristics of the Synthesized Bottlebrush Polymers^a^

**Polymer**	***N***_**BB**_	***N***_**SC1**_	***N***_**SC2**_	***x***	***x*′**	*M*_n_/kDa (NMR)
**PPz**_**BB**_**-PDMS**_**SC**_**-1**	24	26	-	0.2	-	13
**PPz**_**BB**_**-PDMS**_**SC**_**-2**	25	26	-	0.6	-	31
**PPz**_**BB**_**-PDMS**_**SC**_**-3**	300^b^	26	-	1.8	-	1000
**PPz**_**BB**_**-PDMS**_**SC**_**-4**	275	26	-	0.6	-	385
**DMS-H25**	200	-	-	-	-	14
**PDMS**_**BB**_**-PNF**_**SC**_**- PDMS**_**SC**_**-1**	50	-	25	-	0.1	13
**PDMS**_**BB**_**-PNF**_**SC**_**- PDMS**_**SC**_**-2**	50	80	25	0.15	0.25	29
**PDMS**_**BB**_**-PDMS**_**SC**_**-1**	50	80	-	0.4	-	23
**SMS-142**	50	-	-	-	-	4

a Values measured by ^1^H NMR spectroscopy. *N*_BB_ = chain length of the backbone, *N*_SC1_ = chain length of the PDMS side chain, *N*_SC2_ = chain length of the PNF side chain, *M*_n_ = molecular weight calculated by ^1^H NMR spectroscopy.

b Estimated as the repeat unit could not be determined by ^1^H NMR spectroscopy due to the low intensity of the backbone end group. *x* refers to PDMS grafting and *x*′ to the PNF grafting (see Figure 2a).

The PDMS grafting (*x*) was estimated by ^1^H NMR spectroscopy and calculated to have values between 0.2 and 1.8 for the **PPz**_**BB**_**-PDMS**_**SC**_ series (Table 1). Overall a series of bottlebrush polymers was synthesized, varying in their architectural parameters (Table 1). For example, **PPz**_**BB**_**-PDMS**_**SC**_**-4** was prepared with a PPz backbone chain length of 275 repeat units and *N*_SC_ 26. These were subsequently studied for the impact of their structure on the viscoelastic properties, in particular the storage modulus (vide infra), of formed supersoft, solvent-free elastomers.

Moreover, a second series of bottlebrush polymers based on a PDMS backbone with both PNF and PDMS as the side chains was prepared, denoted **PDMS**_**BB**_**-PNF**_**SC**_**-PDMS**_**SC**_**-1–2** (see Figure 2a). To achieve this, styrene-capped [NPCl_2_]_*n*_ was synthesized according to our previous reports, using diphenylphosphinostyrene instead of PPh_3_Cl_2_ as the initiator,^28,29^ and functionalized with a 1:1 ratio of trifluoroethanol (TFE) and 2,2,3,3,4,4,5,5-octafluor-1-pentanol (OFP). Again, the conversion from monomer to polymer was tracked by ^31^P NMR spectroscopy. It showed a complete substitution of the chloride atoms and 25 repeat units, resulting in a PPz of *M*_n_ = 9500 g mol^–1^, denoted as PNF (Figure SI-4). The combination of TFE and OFP was already used in commercial polyphosphazene elastomers for denture liners, which are anecdotally noted for their softness compared to silicone-based denture liners,^30^ and hence was chosen as a starting point for our study. The TFE and OFP with molecular weights of ∼99 and ∼230 g mol^–1^, respectively, are considered as side chains of **PNF**_**SC**_, with densely grafted structure, *N*_g2_ = 0.5. The final **PDMS**_**BB**_**-PNF**_**SC**_**-PDMS**_**SC**_**-1–2** bottlebrush polymers were then constructed based on a commercially available mercapto functionalized PDMS backbone (*N*_BB_ = 50) onto which the side chains were grafted via the vinyl-functionalities using ethyl (2,4,6-trimethylbenzoyl) phenylphosphinate (TPO-L) as a photoinitiator (Figure 2a). The thiolene reaction was characterized by ^1^H NMR spectroscopy, with a quantitative conversion of vinyl groups reacted (Figure 2b). As side chains, both the PNF and a commercial vinyl-end-capped PDMS (*N*_SC_ = 80) were used, as well as a combination of both. Analogous to the **PPz**_**BB**_**-PDMS**_**SC**_ bottlebrush polymers, the viscoelastic properties of the formulated elastomers were investigated. Furthermore, a polymer **PDMS**_**BB**_**-PDMS**_**SC**_**-1** with only PDMS side chains (*N*_sc1_) but the same grafting density was synthesized to subsequently study the influence of the PNF side chain (*N*_sc2_).

**Figure 2:**
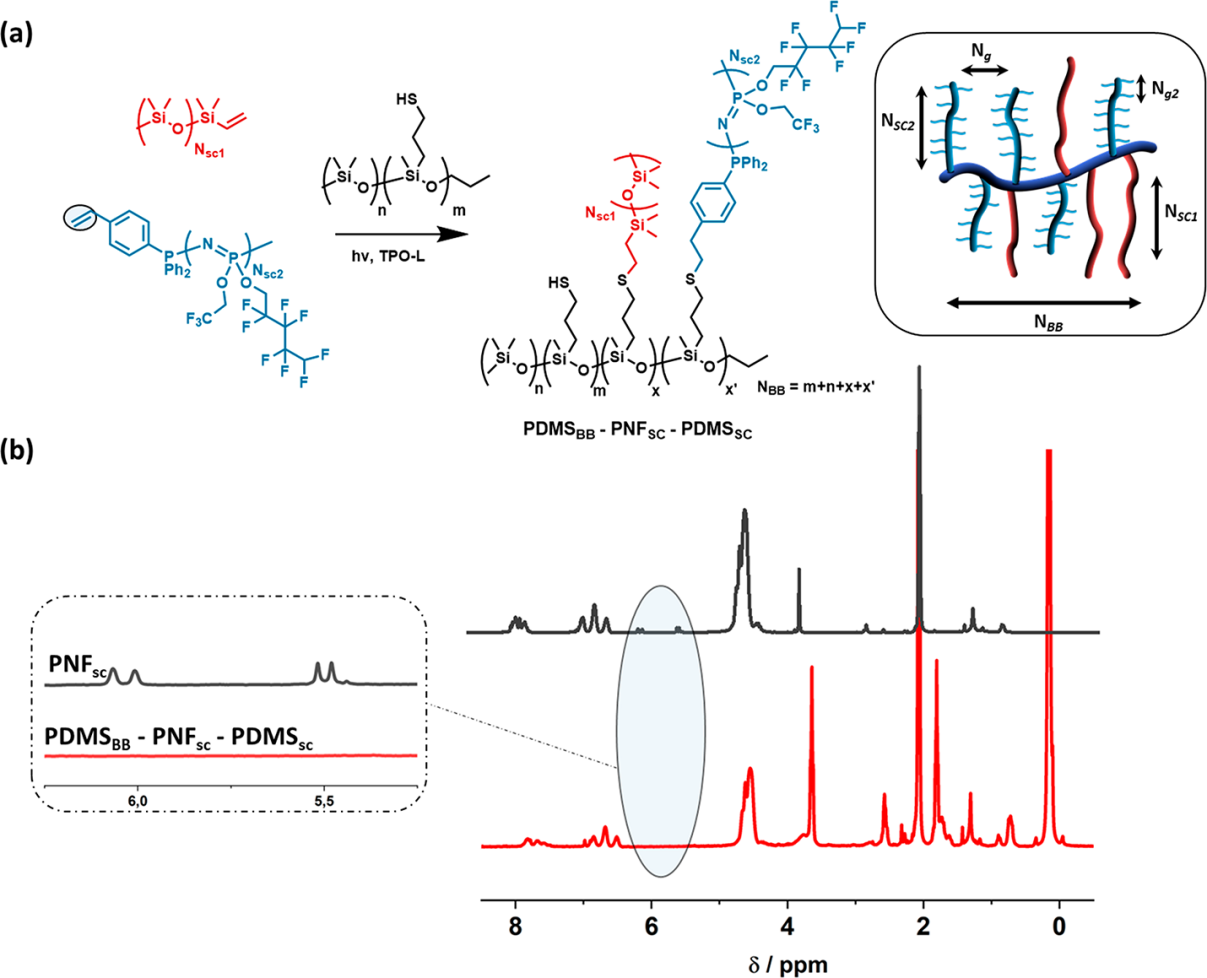
(a) Synthesis of **PDMS**_**BB**_**-PNF**_**SC**_**-PDMS**_**SC**_ polymers with two different types of side chains, PDMS (*N*_SC1_) and PNF (*N*_SC2_), on a PDMS backbone. (b) ^1^H NMR spectra of the individual PPz side chain and **PDMS**_**BB**_**-PNF**_**SC**_**-PDMS**_**SC**_ comb polymer, showing the successful grafting onto the PDMS backbone indicated by the disappearance of the signals of the double bonds (the inset shows the consumption of the styrene end groups upon grafting).

### Elastomers

**Figure 3:**
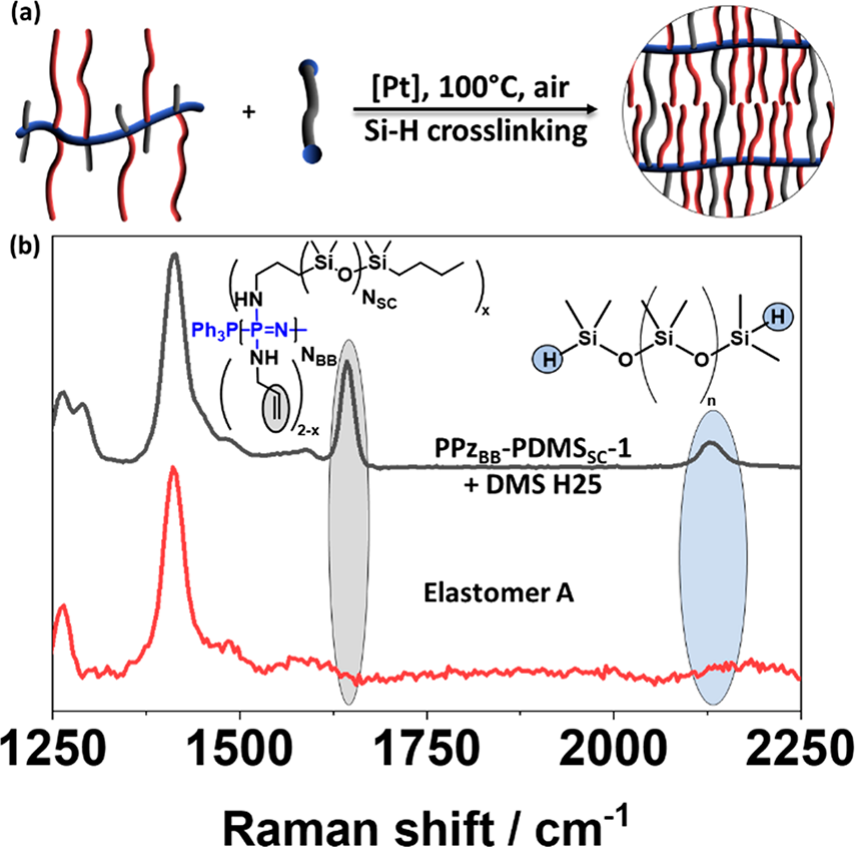
(a) Schematic of the platinum-catalyzed cross-linking and (b) Raman spectra of elastomer **A** and its formulation before cross-linking, showing the disappearance of the hydride- and vinyl-associated peaks.

With this toolbox of bottlebrush polymers in hand, we prepared a series of cured elastomers, as summarized in Table 2. We utilized Karstedt’s (platinum-divinyltetramethyldisiloxane) catalyzed curing of the allyl-moieties with a slight excess of dihydride cross-linker PDMS (**DMS-H25**, *N*_CL_ ≈ 200 repeat units) to cure the bottlebrush polymers. For **PPz**_**BB**_**-PDMS**_**SC**_**-1**, with a relatively low PDMS grafting (*x* = 0.2, *N*_BB_ = 24, *N*_SC_ = 26), quantitative conversion of the allyl groups on the PPz backbone was achieved. This was confirmed by Raman spectroscopy, showing a simultaneous disappearance of the peaks at 1643 cm^–1^ and at 2130 cm^–1^, associated with the allyl groups and the Si–H group, verifying the curing reaction (Figure 3).

**Table 2:** Elastomer Series Prepared with **PPz**_**BB**_**-PDMS**_**SC**_ Polymers and Cured with a Telechelic Si–H Functional PDMS Crosslinker (**DMS-H25**, *N*_CL_ = 200) in the Presence of a Platinum–Divinyltetramethyldisiloxane Complex^a^

**Elastomer**	**Polymer**	**Molar Ratio(Polymer:Cross-linker)**	Storage Modulus/kPa
**A**	**PPz_BB_-PDMS_SC_-1**	45:55	13
**B**	**PPz_BB_-PDMS_SC_-3**	15:85	34
**C**	**PPz_BB_-PDMS_SC_-3**	10:90	46
Ref_DMS-H25	**DMS-H25**	0:100	111

a The shear storage modulus was taken as the measured value at an oscillation frequency of 0.1 rad/s and a temperature of 24 °C.

Unfortunately, **PPz**_**BB**_**-PDMS**_**SC**_**-3**, with a large number of grafted PDMS side-chains (*x* = 1.8, *N*_BB_ = 275, *N*_SC_ = 26), did not cure by this method, presumably due to the low accessibility of the allyl groups on this highly grafted polymer. Therefore, an oxidation reaction recently reported by Skov^31^ was used in which hydrosilanes were cured with themselves in the presence of moisture, oxygen, and a platinum catalyst. PDMS **DMS-H25** (*N*_CL_ ≈ 200) was again used as a cross-linker, as for all other formulations. The successful covalent incorporation of our novel **PPz**_**BB**_**-PDMS**_**SC**_**-3** bottlebrush structures into this formulation was ensured by a large excess of Si–H groups compared to the available allyl-functionalities on the PPz backbone as well as the much faster reaction rate for the hydrosilylation reaction then for the oxidation of hydrosilanes.^31^ As reference material, the cross-linker was reacted with itself, according to the literature procedure described above, resulting in **Ref_DMS-H25**.

**Figure 4:**
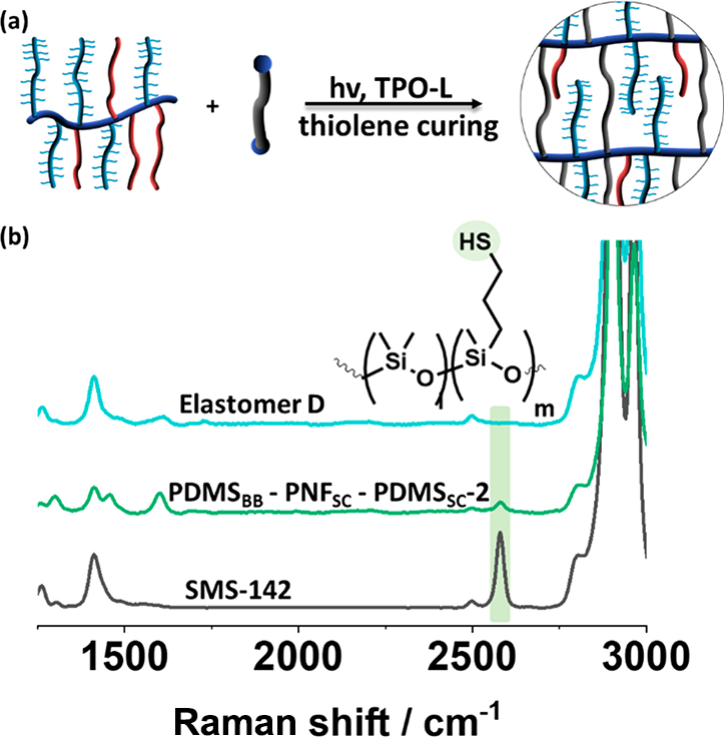
(a) Schematic of thiolene curing of **PDMS**_**BB**_**-PNF**_**SC**_**-PDMS**_**SC**_ polymers and (b) Raman spectra of elastomer **D** and its formulation before cross-linking, showing the disappearance of the thiol groups.

The bottlebrush polymers based on a PDMS backbone (**PDMS**_**BB**_**-PNF**_**SC**_**-PDMS**_**SC**_**-1–2**) were cured by thiolene curing as reported by Liu and co-workers.^32^ The polymers were mixed with the respective commercially available divinyl terminated polydimethylsiloxane DMS V31 (*N*_CL_ ≈ 230) and the photoinitiator TPO-L in a predetermined molar ratio, as shown in Table 3, and exposed to UV light at 365 nm. The conversion was evidenced by Raman spectroscopy (Figure 4) in the absence of any thiol functionality. For all elastomers, either based on **PPz**_**BB**_**-PDMS**_**SC**_ or **PDMS**_**BB**_**-PNF**_**SC**_**-PDMS**_**SC**_, gel fractions were measured to be between 60 and 83% (Table SI-1).

**Table 3:** Elastomers Prepared by Stoichiometric Thiolene Curing with the Divinyl Terminated Polydimethylsiloxane (DMS V31) and TPO-L as a Photoinitiator^a^

**Elastomer**	**Polymer**	Storage Modulus/kPa
**D**	**PDMS_BB_-PNF_SC_-PDMS_SC_-2**	12
**E**	**PDMS_BB_-PDMS_SC_-1**	42
**F**	**SMS-142**	43

a The shear storage modulus was taken as the measured value at an oscillation frequency of 0.1 rad/s and a temperature of 24 °C.

### Rheological Studies

**Figure 5:**
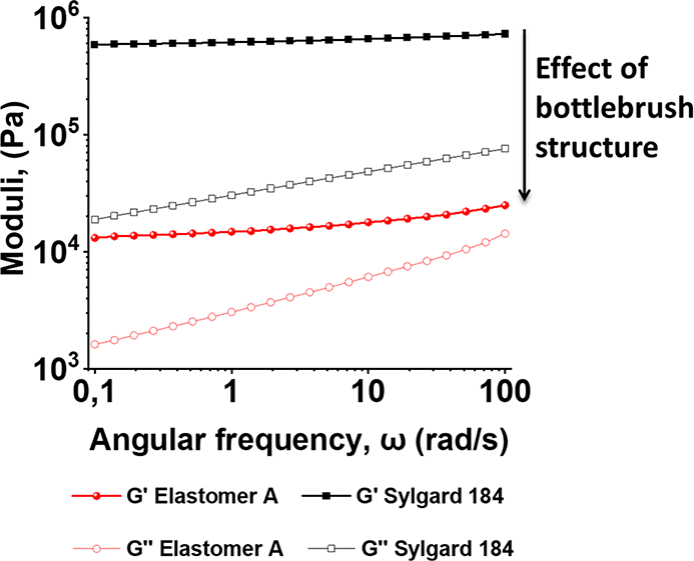
Frequency sweeps of elastomer **A**, and Sylgard 184 as reference, showing that the modulus is shifted downward due to the effect of bottlebrush structure.

The effect of the bottlebrush architecture on the properties of the elastomers (after extensive washing in CH_2_Cl_2_ to remove any non-cross-linked polymers) is shown in Figure 5. Elastomer **A**, synthesized from **PPz**_**BB**_**-PDMS**_**SC**_**-1** (*N*_BB_ = 24, *N*_SC_ = 26, *x* = 0.2) and **DMS-H25** (*N*_CL_ ≈ 200), showed a supersoft elastomer behavior with a rubber-like plateau modulus in the region of 10^4^ Pa, considerably below that of the commercial PDMS Sylgard 184. The rubber-like behavior, or *G*′ secondary plateau, across the full range (up to 100 rad/s) of elastomer **A** is clear evidence of a cured system.

**Figure 6:**
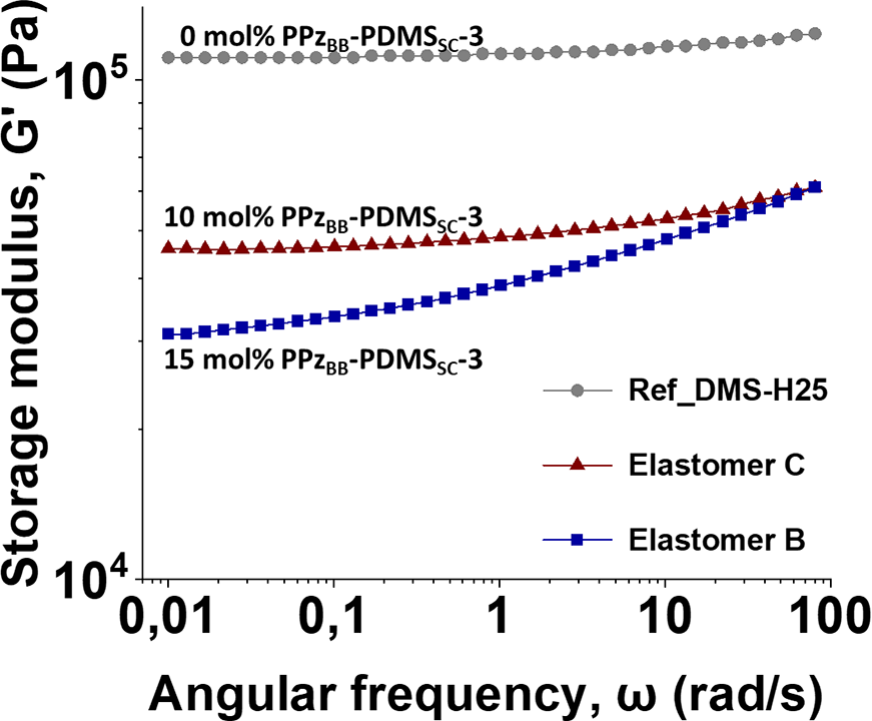
Frequency sweeps of elastomers **B** and **C** showing a decrease in moduli with increasing molar fraction of **PPz**_**BB**_**-PDMS**_**SC**_**-3**, compared with the reference material **Ref_DMS-H25**.

For elastomers **B** and **C**, cured with a large excess of PDMS dihydride, rheological investigations showed that the content of the **PPz**_**BB**_**-PDMS**_**SC**_ bottlebrush is decisive for the softness when compared to **Ref_DMS-H25**. Elastomer **C**, with the highest amount (15%) of **PPz**_**BB**_**-PDMS**_**SC**_**-3**, was observed to be the softest elastomer and a rheologically viscoelastic material without rubber-like behavior (Figure 6). Meanwhile, elastomer **B**, with 10% **PPz**_**BB**_**-PDMS**_**SC**_**-3**, showed an intermediate modulus with a tendency to rubber-like behavior as can be seen in the low frequency range (Figure 6). Unfortunately, it was not possible to prepare elastomers with higher quantities of **PPz**_**BB**_**-PDMS**_**SC**_**-3** as these did not cure sufficiently.

As it is impractical to wash elastomers in real-life applications, we then also studied the rheological properties without washing (Figure SI-5). As expected, the moduli were lower before washing due to the sol fraction. Nevertheless, all trends remained in the structure–property relationships, thus demonstrating that the chemical architecture is the dominant effect on the elastomer properties.

**Figure 7:**
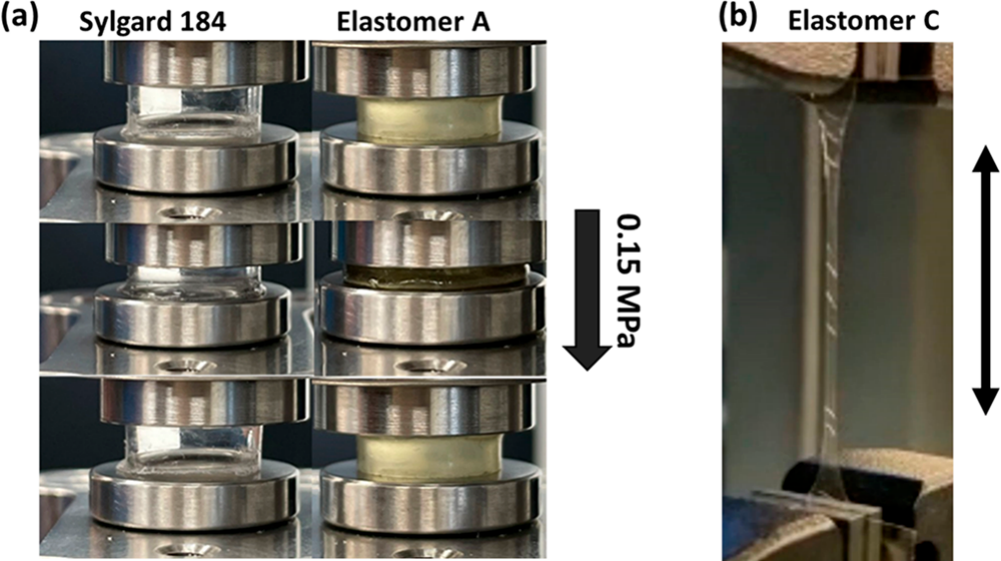
(a) Compression test of the extremely soft elastomer **A**, being compressed to a strain of 70% with a pressure of 0.15 MPa and returning to its original shape after the pressure is released. In contrast, Sylgard 184 can only be compressed to 30%, and the (b) tensile test shows the robust and elastic elastomer **C**.

Commercial PDMS elastomers are often hampered by poor tensile properties, which is classically remedied through the addition of fillers. However, this approach usually results in an increase in modulus.^33^ An exemplary tensile measurement can be seen in Figure 7b showing the elongation of elastomer **C** up to 360% (Figure SI-6). Furthermore, Figure 7a shows the compressibility of elastomer **A** compared to the conventional elastomer Sylgard 184, both subjected to a pressure of 0.15 MPa. Elastomer **A** allows compression to a strain of 70%, whereas Sylgard 184 allows compression to only 30% (Figure SI-7). Despite the high compressive stress, our bottlebrush elastomer **A** returns to its original shape immediately after the pressure is released, showing good elasticity compared to conventional soft elastomers, which often only partially recover.^31^

The second set of elastomers (elastomers **D**–**F**), based on the series of **PDMS**_**BB**_**-PNF**_**SC**_**-PDMS**_**SC**_**1–2** bottlebrush polymers, also showed interesting rheological properties. Even with a low grafting density, relatively soft polymers could be attained when compared to the references Sylgard-184 as well as the thiolene-cured PDMS without any brush content (elastomer **F**, Figure SI-8). Elastomer **D**, synthesized from **PDMS**_**BB**_**-PNF**_**SC**_**-PDMS**_**SC**_**-2** with PNF content, showed the lowest storage modulus with the shortest rubber-like plateau. In contrast, elastomer **E**, with only PDMS side chains and the same *N*_g_ as elastomer **D** but no PNF side chains, exhibited a significantly higher modulus compared to elastomer **E**, including the broadest rubber-like plateau.

Taken together, these results show that the observed softness for PNF containing polymers is an effect of the incorporated PNF and the bottlebrush structure. We postulate that the reason for this is its flexible molecular structure and its brush-on-brush type architecture (*N*_g2_ = 0.5), which introduces considerably more “dangling ends” into the system (Figure 4a). Indeed, overall for the different series in this study, the data point to *N*_g_ is the most dominant effect on elastomer softness.

### Energy Dissipating Behavior

Such free “dangling” chain-ends and the relative motion attributed to them has been established in synthetic damping elastomers as a method to increase their energy dissipation.^34^ The rheological data of the elastomers based on our **PPz**_**BB**_**-PDMS**_**SC**_ bottlebrush series (elastomers **A**–**C**) indeed show increased tan δ values and thus higher energy dissipation capabilities compared to a non-bottlebrush PDMS elastomer used as a reference (Figure SI-9). The elastomers formulated on the **PDMS**_**BB**_**-PNF**_**SC**_**-PDMS**_**SC**_ series, with PNF as side chains, showed large tan δ values, even with relatively low grafting densities (Figure SI-10).

**Figure 8:**
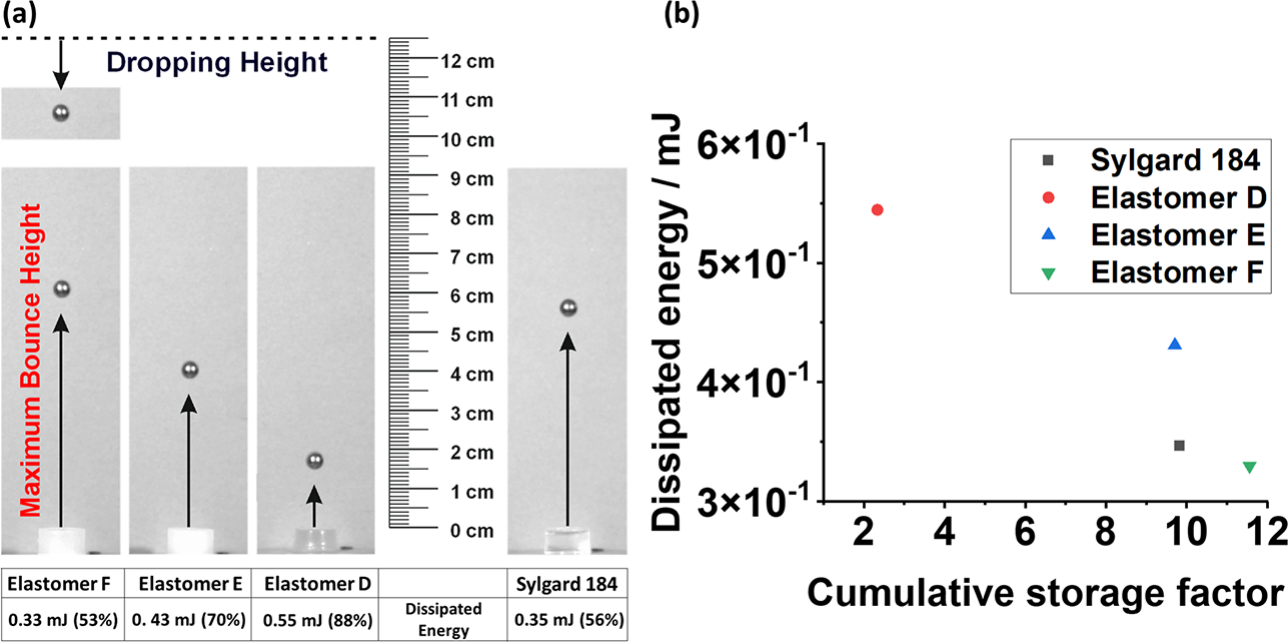
Ball drop experiments assessing the energy dissipation potential of elastomers **D**–**F**. (a) At the right, the experimental setup can be seen. The materials were tested by dropping a steel ball (*d* = 5 mm, 0.504 g) from a height of 125 mm and measuring the maximum bounce height with a high-speed camera. (b) The graph on the left shows the energy dissipated by the materials over the cumulative storage factor.

Based on these findings, the energy dissipation abilities of elastomers based on the **PDMS**_**BB**_**-PNF**_**SC**_**-PDMS**_**SC**_ polymers were further tested by means of a ball drop test^35^ (Figure 8). For the ball drop test, steel balls (*d* = 5 mm, 0.504 g) were dropped from a height of 125 mm onto the sample pellets and the bounce height to which they bounced back up was measured by means of a high-speed camera (see the videos in the Supporting Information). The dissipated energy of the respective sample was calculated from the difference in potential energy of the steel ball at the dropping height and maximum bounce height (Figure 8a). Elastomer **F**, containing no bottlebrush polymers, showed the lowest damping behavior, with a dissipated energy similar to that of commercial Sylgard 184. Meanwhile, elastomer **D**, containing **PDMS**_**BB**_**-PNF**_**SC**_**-PDMS**_**SC**_**-2**, absorbed the most energy. In contrast, a sample with the same brush content as elastomer **D** but only PDMS side chains (elastomer **E**) dissipated energy to a degree approximately between that of elastomers **F** and **D**, nicely illustrating the crucial effect of the PNF side chains on the energy dissipation of these materials.

To facilitate the direct comparison of the synthesized complex polymer systems, the so-called cumulative storage factor (CSF) and cumulative complex viscosity (CCV) were calculated and plotted against each other (Figure SI-11). This enables a comparison of the rigidity (level of interactions) within the system directly based on information from the loss factor curves.^36^ As can be seen from Figure SI-11a, the highest level of rigidity (highest CSF) and lowest level of mobility (highest CCV) were observed for **Ref_DMS-H25**. Elastomers **A**–**C** showed significantly lower CSF and CCV values, reflecting a higher level of damping and mobility. Comparing elastomer series **D**–**F** (Figure SI-11b), the highest level of damping and mobility (lowest CSF and CCV values, respectively) was observed for elastomer **D**. On the other hand, the highest level of rigidity was seen for sample **F**, without any PNF and brush content. As can be seen for elastomer **E**, PDMS brushes improve these properties, but by far not as much as the formulations with the PNF components, which is mainly due to the additional tangling ends on the brush-on-brush type architecture. In addition, plotting the dissipated energy results from the ball drop test against the calculated CSF values shows an excellent correlation of these values for the characterization of damping behavior (Figure 8b). As already displayed by the previous result, this makes it clear that the highest damping correlates with the PNF content.

## Conclusion

An inorganic bottlebrush polymer platform has been developed based on a combination of polydimethylsiloxane and polyphosphazene chemistry in a controlled synthesis route. This unique approach allows facile adjustment of the structural features of the bottlebrush polymers to exploit the distinctive properties of these polymers in cured elastomers. The rheological properties of these elastomers showed good elasticity and compressibility in combination with low moduli comparable to those of hydrogels and human soft tissue. Furthermore, by grafting PPz onto PDMS, the number of dangling ends could be further increased, resulting in materials with significantly higher energy dissipation than PDMS-based elastomers, as demonstrated by both oscillatory rheometry and ball drop tests. With a clear correlation between molecular design and mechanical properties, this research presents a robust synthetic platform for tailored, solvent-free supersoft elastomers.

## Materials and Methods

Chemicals were purchased from different commercial providers and used as received if not specified any differently. All polydimethylsiloxanes used were from Gelest and are listed in Table SI-2. Platinum(0) 1,3-diethenyl-1,1,3,3-tetramethyldisiloxane complexes (Karstedt’s catalyst, ∼2% Pt in xylene), allylamine, N-Boc-allylamine, lithium bis(trimethylsilyl)amide (LiN(SiMe_3_)_2_), phosphorus trichloride, thionyl chloride, 4-(diphenylphosphino)styrene, dichlorotriphenylphosphorane (Ph_3_PCl_2_), 2,2,2-trifluoroethanol (TFE), and 2,2,3,3,4,4,5,5-octafluor-1-pentanol (OFP) were purchased from Sigma-Aldrich. Ethyl acetate, dichloromethane (DCM), toluene, magnesium sulfate (MgSO_4_), and sodium hydrogen carbonate (Na_2_CO_3_) were purchased from VWR, anhydrous dichloromethane, tetrahydrofuran (THF), and Celite (325 mesh powder) were purchased from Alfa Aesar, triethylamine was purchased from Merck, trifluoroacetic acid (TFA) was purchased from Fluka, ethyl (2,4,6-trimethylbenzoyl)-phenylphosphinate (TPO-L) was purchased from Fluorochem, and chloroform-*d*, deuterated acetone, and deuterated methanol were purchased from Eurisotop. Polymer synthesis and modification were carried out under inert conditions using a glovebox (MBRAUN) with an argon atmosphere. For the monomer synthesis, all glassware and Celite was dried in an oven overnight at 120 °C prior to use. Triethylamine was distilled and stored over molecular sieves (3 Å) prior to use. The Raman measurements were performed on a WITEC Alpha 300 R-Raman system. The laser was a Nd:YAG with a wavelength of 532 nm, and the laser intensity for the measurements was 15 mW. The spectra were recorded from 150 to 3400 cm^–1^. Photochemical reactions were carried out at 5 °C in a Rayonet chamber reactor with a CAMAG UV-lamp centered at 365 nm. ^1^H, ^31^P, and ^29^Si NMR spectroscopy were performed on a Bruker 300 MHz spectrometer and referenced to the signal of internal CDCl_3_, (CD_3_)_2_CO and MeOD. ^1^H NMR spectral data are given in δ/ppm relative to residual solvent peaks. ^31^P NMR spectral data at 121 MHz are given in δ/ppm relative to an external standard of 85% phosphoric acid.

## Synthesis

### Synthesis of Trichlorophosphoranimine (Cl_3_PNSiMe_3_)

The trichlorophosphoranimine monomer was synthesized using a similar approach to the literature.^37^ LiN(SiMe_3_)_2_ (24.95 g, 0.15 mol, 1 equiv) was dissolved in 500 mL of anhydrous diethyl ether under argon and cooled to 0 °C. Phosphorus trichloride (13.05 mL, 0.15 mol, 1 equiv) was added dropwise to the stirred solution within 20 min. The reaction was kept at 0 °C for 30 min and stirred for another period of 30 min at room temperature. Afterward, sulfuryl chloride (12.05 mL, 0.15 mol, 1 equiv) was added dropwise over 20 min and the reaction mixture was again stirred as before. Then, the reaction mixture was filtered through dry Celite and the solvent was removed under reduced pressure. The final product was obtained via vacuum distillation at 4 mbar and 41 °C as a clear and colorless liquid (20.01 g, yield 61%). The monomer Cl_3_PNSi(CH_3_)_3_ was stored under argon at −35 °C.

^1^H NMR (300 MHz, CDCl_3_, δ): 0.18 ppm (s, 9H); ^31^P NMR (121 MHz, CDCl_3_, δ): −54.44 ppm.

### Bottlebrush Polymer Series **PPz_BB_-PDMS_SC_-1–4**

The poly(dichloro)phosphazene (NPCl_2_) was synthesized with modifications from literature procedures.^27^ The synthesis was carried out in the glovebox under an argon atmosphere at room temperature. The phosphine mediator Ph_3_PCl_2_ (8 mg, 0.023 mmol, 1 equiv) and the monomer Cl_3_PNSi(CH_3_)_3_ (135 mg, 0.6 mmol, 25 equiv) were dissolved separately in about 0.5 mL of dichloromethane. Then, the monomer solution was added dropwise to the Ph_3_PCl_2_ solution, and the mixture was stirred for 12 h. The resulting product (quantitative yield) was confirmed via ^31^P NMR spectroscopy and used without any further purification for the macrosubstitution.

To obtain a range of polymers with different brush contents, different ratios of commercial monoaminopropyl terminated polydimethylsiloxanes MCR-A12 were used. In the following, the synthesis of **PPz**_**BB**_**-PDMS**_**SC**_**-2** (*x* = 0.6) is described as an example. The functionalized PDMS (3.1 g, 1.6 mmol, 0.6 equiv) and triethylamine (0.9 mL, 6.7 mmol, 2.5 equiv) were dissolved in anhydrous THF and added dropwise to the poly(dichloro)phosphazene in 20 mL of THF (0.61 g, 2.6 mmol, 1 equiv), resulting in an exothermic reaction where a white precipitate was formed. After stirring the reaction mixture for 16 h, allylamine (0.3 mL, 5 mmol, 1.9 equiv) was added and the mixture was stirred for a further 16 h. The formed precipitate was removed by filtration, and the solvent was evaporated under reduced pressure. The mixture was redissolved in 20 mL of ethyl acetate and washed with brine twice. To separate the phases, it was necessary to centrifuge the solution at 5500 rpm for 10 min. The organic phase was dried with MgSO_4_ and the solvent was removed under a vacuum after filtration, resulting in a yellowish viscous product. The determination of the repeat units (targeted 25 and 300) was based on the ^1^H NMR spectra using the aromatic PPz-backbone end group (Figure SI-1).

#### NPCl_2_

^31^P NMR (121 MHz, CDCl_3_, δ): −18.20, 20.04 ppm.

#### **PPz_BB_-PDMS_SC_-1**

Yield: 61%; ^1^H NMR (300 MHz, CDCl_3_, δ): 7.83–7.40 (m, 15H), 6.10–5.74 (m, 42H), 5.32–4.88 (m, 84H), 3.51 (s, 84H), 1.85–1.62 (m, 10H), 1.39–1.23 (m, 20H), 0.95–0.80 (m, 15H), 0.54 (t, 20H), 0.20–(−0.15) (m, 785H) ppm; ^31^P NMR (121 MHz, CDCl_3_, δ): 16.62, 10.41, 3.10 ppm; ^29^Si NMR (60 MHz, CDCl_3_, δ): 17.41, 7.60, (−21.32)–(−22.56) ppm.

#### **PPz_BB_-PDMS_SC_-2**

Yield: 60%; ^1^H NMR (300 MHz, CDCl_3_, δ): 7.78–7.47 (m, 15H), 6.02–5.79 (m, 26H), 5.34–4.85 (m, 52H), 3.52 (s, 52H), 1.87–1.65 (m, 23H), 1.41–1.21 (m, 45H), 0.90–0.82 (m, 34H), 0.54 (t, 45H), 0.31–(−0.15) (m, 1767H) ppm; ^31^P NMR (121 MHz, CDCl_3_, δ): 18.10, 9.95, 2.91 ppm; ^29^Si NMR (60 MHz, CDCl_3_, δ): 7.56, (−21.54)–(−22.60) ppm.

#### **PPz_BB_-PDMS_SC_-3**

Yield: 67%; ^1^H NMR (300 MHz, CDCl_3_, δ): 7.78–7.50 (m, 15H), 5.91 (m, 1H), 5.23–4.95 (m, 2H), 3.55 (s, 2H), 1.93–1.67 (m, 2H), 1.41–1.21 (m, 4H), 0.84–0.78 (m, 3H), 0.54 (t, 4H), 0.35–(−0.15) (m, 156H) ppm; ^31^P NMR (121 MHz, CDCl_3_, δ): 12.25, 2.35 ppm; ^29^Si NMR (60 MHz, CDCl_3_, δ): 7.59, −21.96 ppm.

#### **PPz_BB_-PDMS_SC_-4**

Yield: 71%; ^1^H NMR (300 MHz, CDCl_3_, δ): 7.84–7.48 (m, 15H), 5.89 (m, 353H), 5.19–4.97 (m, 706H), 3.52 (s, 710H), 1.99–1.72 (m, 386H), 1.41–1.21 (m, 772H), 0.95–0.80 (m, 580H), 0.54 (t, 772H), 0.33–(−0.15) (m, 29600H) ppm; ^31^P NMR (121 MHz, CDCl_3_, δ): 10.67, 3.20 ppm; ^29^Si NMR (60 MHz, CDCl_3_, δ): 7.58, (−21.73)–(−22.15) ppm.

### Synthesis of Poly(fluoroalkoxy)phosphazene (PNF)

The poly(fluoroalkoxy)phosphazene was synthesized according to adapted literature procedures at room temperature in the glovebox under an argon atmosphere.^26,28^ To this end 4-(diphenylphosphino) styrene (100.0 mg, 0.35 mmol, 1 equiv) and hexachloroethane (90.3 mg, 0.38 mmol, 1.1 equiv) were dissolved separately in about 0.5 mL of dichloromethane, mixed, and reacted overnight. Subsequently, the monomer Cl_3_PNSi(CH_3_)_3_ (1.9468 g, 8.67 mmol, 25 equiv) was dissolved in about 2 mL of DCM, added to the reaction solution, and stirred for an additional 24 h. The resulting poly(dichloro)phosphazene was further reacted in a macromolecular substitution reaction without purification.

2,2,2-Trifluoroethanol (1.31 mL, 17.34 mmol, 50 equiv) and 2,2,3,3,4,4,5,5-octafluoro-1-pentanol (2.41 mL, 17.34 mmol, 50 equiv) as macrosubstituents were dissolved in approximately 50 mL of THF, and NaH (1.3872 g, 34.68 mmol, 100 equiv) was added portionwise, avoiding excessive gas evolution. After stirring the reaction mixture overnight, the synthesized [NPCl_2_]_*n*_ was added, resulting in a white precipitate, and the reaction was again stirred for 24 h. Finally, the reaction solution was evaporated to dryness and precipitated in H_2_O (2×) and heptane (3×) from THF. After drying in the vacuum drying oven at 80 °C for 24 h, the final poly(fluoroalkoxy)phosphazene was obtained as a beige, highly viscous oil. The determination of the repeat units (targeted 25) is based on the ^1^H NMR spectra using the aromatic PPz-backbone end group.

Yield: 70%; ^1^H NMR (300 MHz, (CD_3_)_2_CO, δ): 7.88–7.58 (m, 14H), 6.66 (t, 28H), 6.02 (d, 1H), 5.48 (d, 1H), 4.80–4.22 (m, 114H) ppm; ^31^P NMR (121 MHz, (CD_3_)_2_CO, δ): 18.23, −3.39, −7.72 ppm; ^19^F NMR (282 MHz, (CD_3_)_2_CO, δ): −76.09, −121.63, −125.72, −130.61, 139.04, −139.22 ppm.

### Bottlebrush Polymer Series **PDMS_BB_-PNF_SC_-PDMS_SC_-1–2**

As an example, the procedure for **PDMS**_**BB**_**-PNF**_**SC**_**-PDMS**_**SC**_**-2** is described. (Mercaptopropyl)methylsiloxane]-dimethylsiloxane copolymer **SMS-142** (0.15 g, 0.3 mmol, 1 equiv) and PNF (0.63 g, 0.07 mmol, 0.25 equiv) were dissolved in anhydrous THF, and TPO-L (2 wt %) was added. Under argon, the reaction was placed in the UV-chamber at 365 nm and a temperature of 4 °C for 45 min under vigorous stirring. Subsequently, under reduced pressure, the solvent was removed to give a viscous yellow liquid. The complete conversion of the reaction was confirmed via ^1^H NMR spectroscopy, and the polymer was used without any further purification for the next step. To this end, monovinyl terminated polydimethylsiloxane MCR-V21 (0.24 g, 0.04 mmol, 0.15 equiv) was reacted with the product as described for the PNF above. Finally, the solvent was removed to give a viscous yellow liquid. For **PDMS**_**BB**_**-PNF**_**SC**_**-PDMS**_**SC**_**-1** (0.1 equiv of PNF) no additional PDMS was added.

#### **PDMS_BB_-PNF_SC_-PDMS_SC_-1**

Yield quantitative; ^1^H NMR (300 MHz, CDCl_3_, δ): 7.81–7.56 (m, 15H), 6.85–6.45 (t, 25H), 4.53 (br, 94H), 2.54 (s, 23H) 1.80–0.66 (m, 130H), 0.37–(−0.09) (m, 363H) ppm; ^31^P NMR (121 MHz, CDCl_3_, δ): 18.15, (−3.41), (−7.75) ppm.

#### **PDMS_BB_-PNF_SC_-PDMS_SC_-2**

Yield quantitative; ^1^H NMR (300 MHz, CDCl_3_, δ): 7.84–7.46 (m, 30H), 6.84–6.38 (t, 30H), 4.54 (br, 227H), 2.55 (s, 23H), 0.39–(−0.08) (m, 1050H) ppm; ^31^P NMR (121 MHz, CDCl_3_, δ): 18.33, 15.19, (−7.74), (−3.39) ppm.

### Elastomers **A**–**C**

Elastomers were prepared from the synthesized polymers **PPz**_**BB**_**-PDMS**_**SC**_**-1** and **PPz**_**BB**_**-PDMS**_**SC**_**-3** and the hydride terminated polydimethylsiloxane PDMS **DMS-H25** in specific ratios (Table 2). A representative procedure for elastomer **A** was as follows: **PPz**_**BB**_**-PDMS**_**SC**_**-1** (70 mg, 0.08 mmol, 1 equiv) and **DMS-H25** cross-linker (1.6 g, 0.11 mmol, 0.7 equiv) were mixed using a vortex mixer and an ultrasonic bath for 15 min. Then the platinum catalyst was added (2 wt %) to the mixture and again mixed in the same way for another 5 min. This mixture was poured into a mold and placed in an oven at 100 °C for 24 h. For the reference sample **Ref_DMS-H25**, the PDMS was mixed only with the platinium catalyst and poured into a mold and placed in an oven at 100 °C for 24 h. Elastomers were analyzed by Raman spectroscopy. The following exemplary data are for elastomer **A**:

Raman (solid): ν_max_ = 3108 (Si–CH_3_), 2943 (C–H), 1410 (Si–CH_3_), 1264 (P–N), 706 (Si–O–Si) cm^–1^.

### Elastomers **D**–**F**

For elastomers **D**–**F**, polymers **PDMS**_**BB**_**-PNF**_**SC**_**-PDMS**_**SC**_**-2** and **PDMS**_**BB**_**-PDMS**_**SC**_**-1** were mixed with divinyl terminated polydimethylsiloxane PDMS in a predetermined molar ratio as shown in Table 3. In short, based on elastomer **D** as an example: **PDMS**_**BB**_**-PNF**_**SC**_**-PDMS**_**SC**_**-2** (1.01 g, 0.04 mmol, 1 equiv) and DMS V31 (1.11 g, 0.07 mmol, 0.25 equiv) were dissolved in acetone and stirred for 30 s on the vortexer; then the photoinitiator ethyl (2,4,6-trimethylbenzoyl) phenyl phosphinate (2 wt %) was added under exclusion of light and stirred for another 30 s. The solvent was carefully removed under a vacuum, and the mixture was poured into a translucent mold and placed for 3 h in the UV-chamber at 365 nm and a temperature of 4 °C. Selected elastomers were analyzed by Raman spectroscopy. For elastomer **D** as an example, the following data were collected:

Raman (solid): ν_max_ = 3115 (Si–CH_3_), 2964 (C–H), 1411 (Si–CH_3_), 1260 (P–N), 708 (Si–O–Si) cm^–1^.

### Gel Fraction

To determine the gel fractions of the prepared elastomers, samples containing approximately 100 mg (*m*_A_) were placed in roughly 20 mL of CH_2_Cl_2_ for 48 h to remove the unreacted polymers. The swollen samples were filtered through glass sinter crucibles, washed with DCM, and dried at room temperature for 24 h (*m*_B_). The experiment was performed in triplicate, and the gel fraction was calculated by .

### Linear Viscoelasticity (Rheology)

Rheological characterization of the gel samples was conducted through oscillatory tests utilizing a stress-controlled Anton Paar MCR 501 rheometer with a standard plate–plate geometry. Gel samples were trimmed to a diameter of 8 mm to exactly fit the used 8 mm parallel-plate measuring system. The gel sample height varied by 1 mm. Amplitude sweeps with a constant frequency of 1 rad/s and a strain rate γ from 1% up to 1000% were run (exemplary data shown in Figures SI-12 and SI-13), followed by frequency sweeps at a constant rate of deformation (γ = 1%) with an angular frequency ranging from 0.01 up to 100 rad/s. For samples with a prolonged LVE (deformation γ > 10%) frequency sweeps were repeated with deformation rates of 2.5%, 5%, and 10%, yielding comparable results and congruent graphs.

### Uniaxial Tensile Test

Samples were prepared from elastomer films (width, 4.1 mm; length, 9.5 mm; thickness, 1 mm) and used to further investigate mechanical properties such as tensile strength, using a TA Instruments DMA Q800 to carry out controlled force tensile tests. Test sample ends were placed between a stationary upper clamp and a moveable lower clamp. Applied normal force was increased by 0.1 N per 60 s, while sample temperature was held constant at 30 °C within the test chamber.

### Compression Test

Cylindrical elastomer samples were formed (*d* = 8 mm, *h* ≈ 5 mm) and tested for compressibility using a DMA Q800. Compression of the elastomers by two round plates (10 mm diameter) was used. The applied forces and distance between the plates were recorded during compression.

### Energy Dissipation (Ball Drop Experiments)

To investigate energy dissipation properties, samples were prepared by casting about 2.1 g of the respective formulations inside a 10 mL syringe. After curing, the samples with a diameter of 7 mm and a height of 10 mm were removed and subjected to ball drop tests. A steel ball with 5 mm diameter and a weight of 0.504 was dropped from 125 mm height onto the sample, and the rebound height of the ball was measured by means of recording videos with a high-speed camera (Phantom Micro C110, High-Speed Vision). The degree of energy absorption was evaluated by calculating the potential energy of the steel ball at dropping height and maximum bounce height using tracking software (Tracker Physlets V6.1.2). For each sample the experiment was carried out in triplicate (Table SI-3).

## Supporting Information Available

Additional data for polymer characterization (PDF)
Video of the ball drop experiment for elastomer **D** (MP4)
Video of the ball drop experiment for elastomer **E** (MP4)
Video of the ball drop experiment for elastomer **F** (MP4)
Video of the ball drop experiment for Sylgard 184 (MP4)
