# Supplementary material for: Inorganic Bottlebrush and Comb Polymers as a Platform for Supersoft, Solvent-Free Elastomers
Source: ACS Polym Au. 2024 Jan 11;4(1):56–65. doi: 10.1021/acspolymersau.3c00043 (PMC10870749; doi:10.1021/acspolymersau.3c00043)
Supplement: Supplementary file 3 — lg3c00043_si_003.pdf [file lg3c00043_si_003.pdf]

Supporting Information

Inorganic bottlebrush and comb polymers as a platform for supersoft, solvent-free elastomers

*Edip Ajvazi[1,2], Felix Bauer[1], Paul Strasser[1], Oliver Brüggemann[1], Rene Preuer[2], Milan Kracalik[3], Sabine Hild[3], Mahdi Abbasi[4], Ingrid Graz,[2] and Ian Teasdale[1]**

[1] Institute of Polymer Chemistry, Johannes Kepler University Linz, Altenberger Straße 69, 4040 Linz, Austria

[2] Christian Doppler Laboratory for Soft Structures for Vibration Isolation and Impact Protection (ADAPT), School of Education, STEM Education, Johannes Kepler University Linz, Altenberger Straße 69, 4040 Linz, Austria

[3] Institute of Polymer Science, Johannes Kepler University Linz, Altenberger Straße 69, 4040 Linz, Austria

[4] Borealis Polyolefine GmbH, Innovation Headquarters, St.-Peter-Straße 25, 4021, Linz, Austria

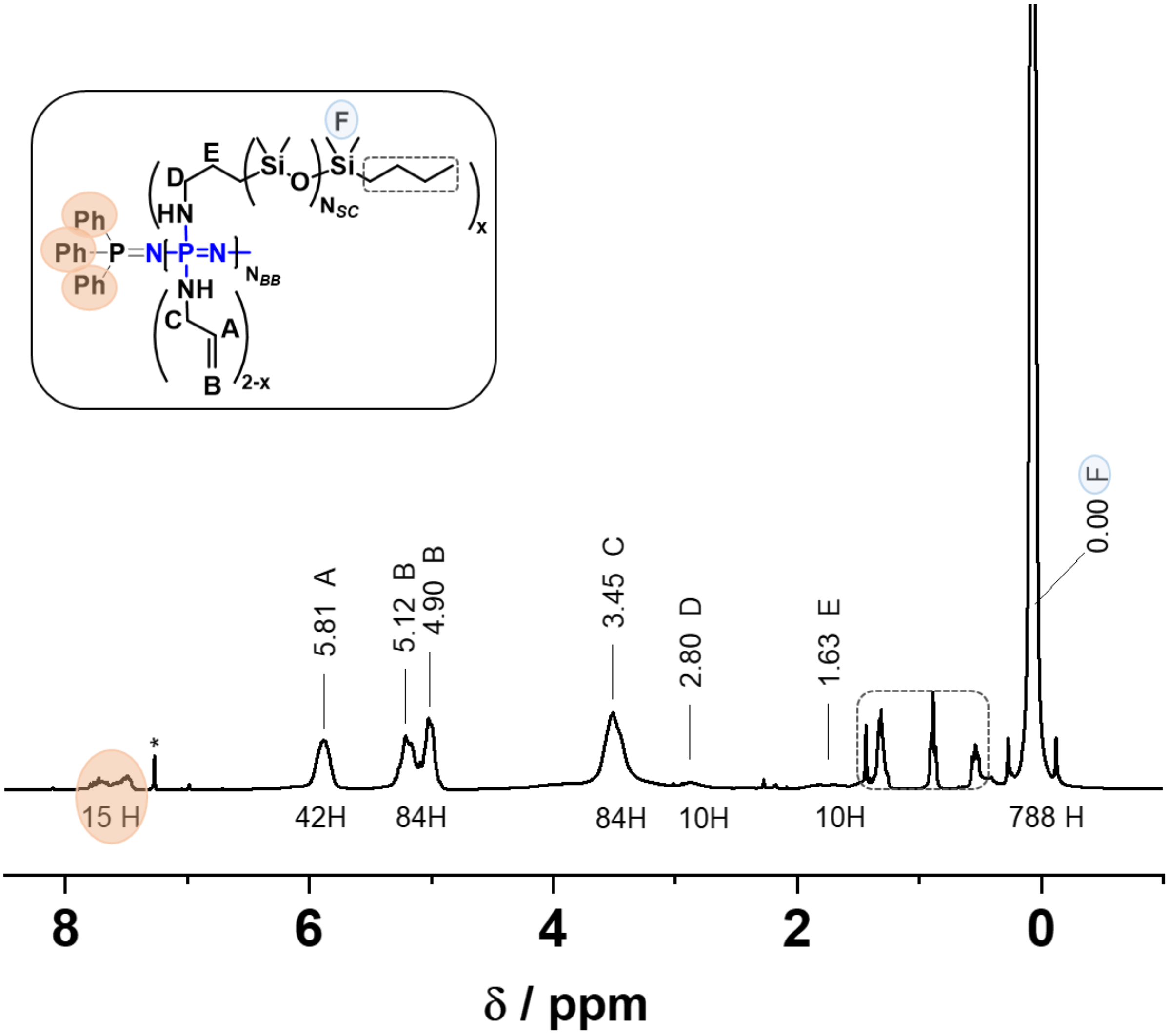

Figure SI-1. ^{1}H NMR spectrum in $CDCl_3$ of the **PPz$_{BB}$-PDMS$_{SC}$-1.** Determination of the number of repeat units was done using the initiator end group. The theoretical grafting density x can be confirmed by the ratio of two integrals of the different side chains.

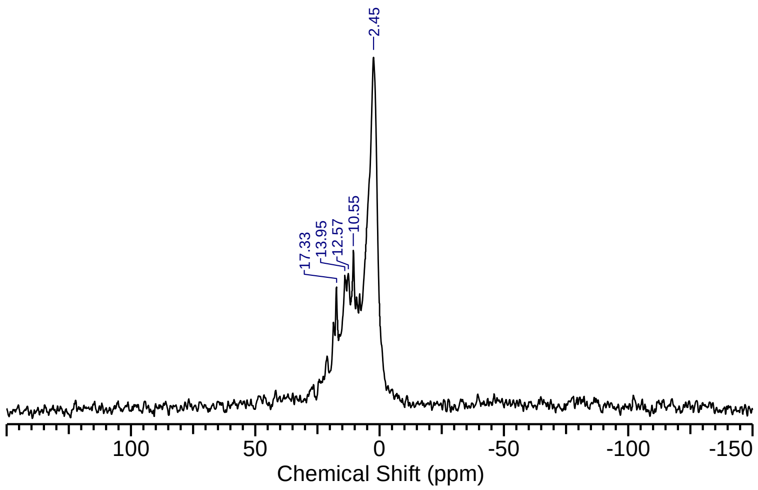

Figure SI-2. ^{31}P-NMR spectrum of **PPzBB-PDMSSC** (N_{SC} = 26) demonstrating complete saturation (x=2) of the PPz backbone with PDMS side chains (MCR-A12).

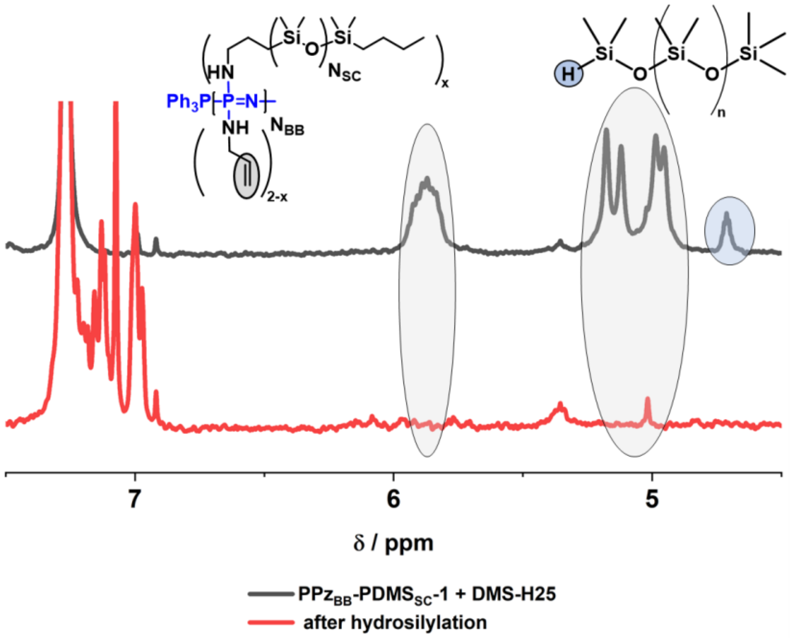

Figure SI-3. ^{1}H NMR spectrum showing a quantitative conversion of double bonds of the allylamine substituents on **PPzBB-PDMSSC-1** and thus confirming their availability for further reactions.

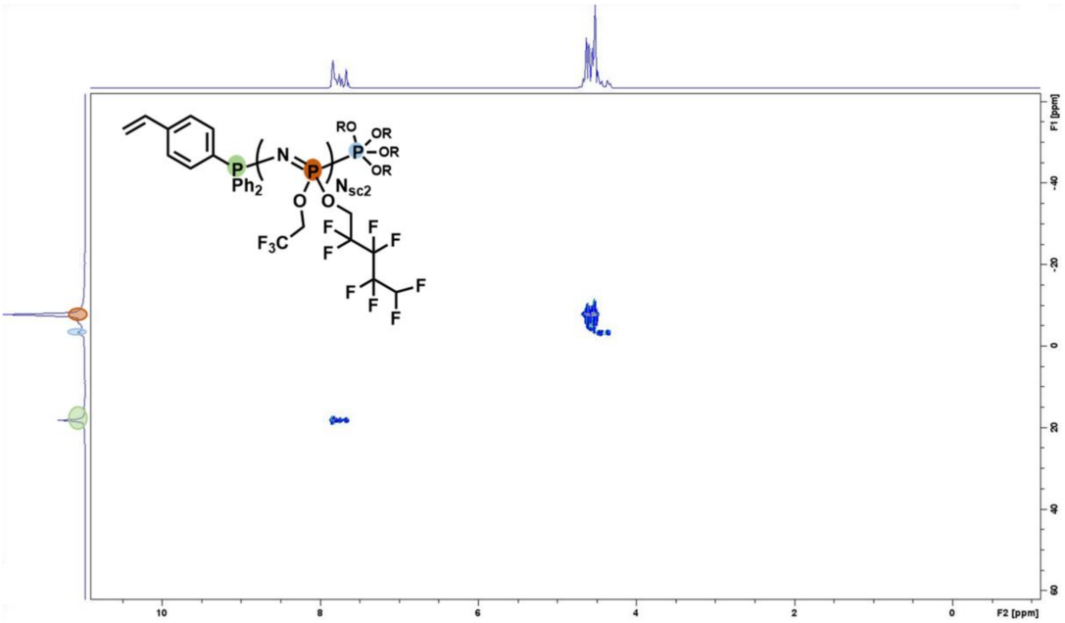

Figure SI-4. ^{1}H-^{31}P-Heteronuclear multiple bond correlation (HMBC) spectrum of the poly(fluoroalkoxy)phosphazene (PNF) used as side chains in **PDMS$_{BB}$-PNF$_{SC}$-PDMS$_{SC}$** bottlebrush polymers. The spectrum confirming a correlation between the aromatic protons and the phosphorus atom which carries the phosphine end group at 18.2 ppm (green). Furthermore, the peak at -7.7 corresponds to the repeating unit in the polymer chain (red) and the signal at -3.4 from the end group at the opposite chain end (blue).

Table SI-1. Gel fractions of the investigated networks.

Elastomers	Gel fractions (%)
A	80
B	77
C	83
D	62
E	60
F	68
Sylgard 184	95
Ref_DMS H25	92

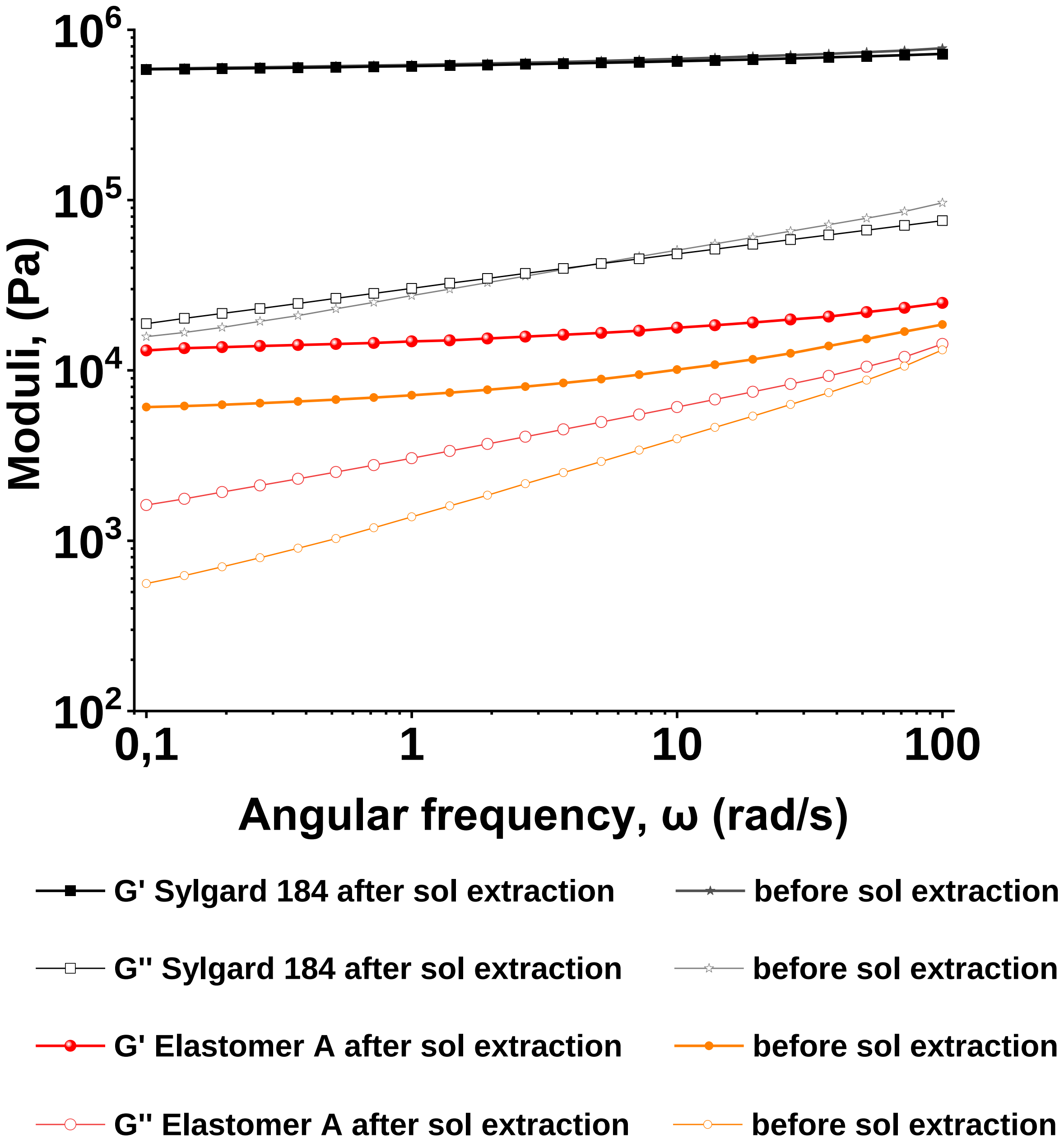

Figure SI-5. Effects of sol fraction on properties of Elastomer **A**.

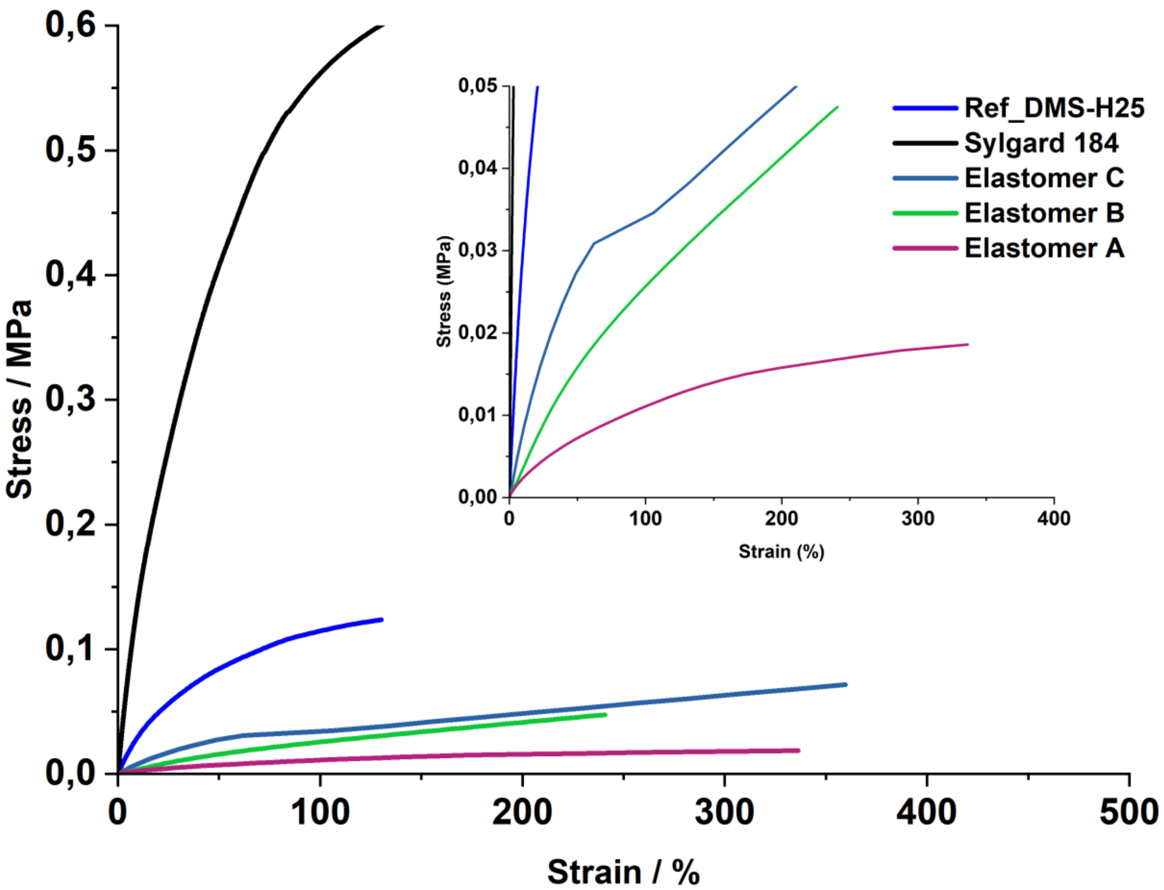

Figure SI-6. Uniaxial tensile tests (Dynamic mechanical analysis) of elastomers **A-C** compared to the reference samples Sylgard 184 and Ref_DMS-H25.

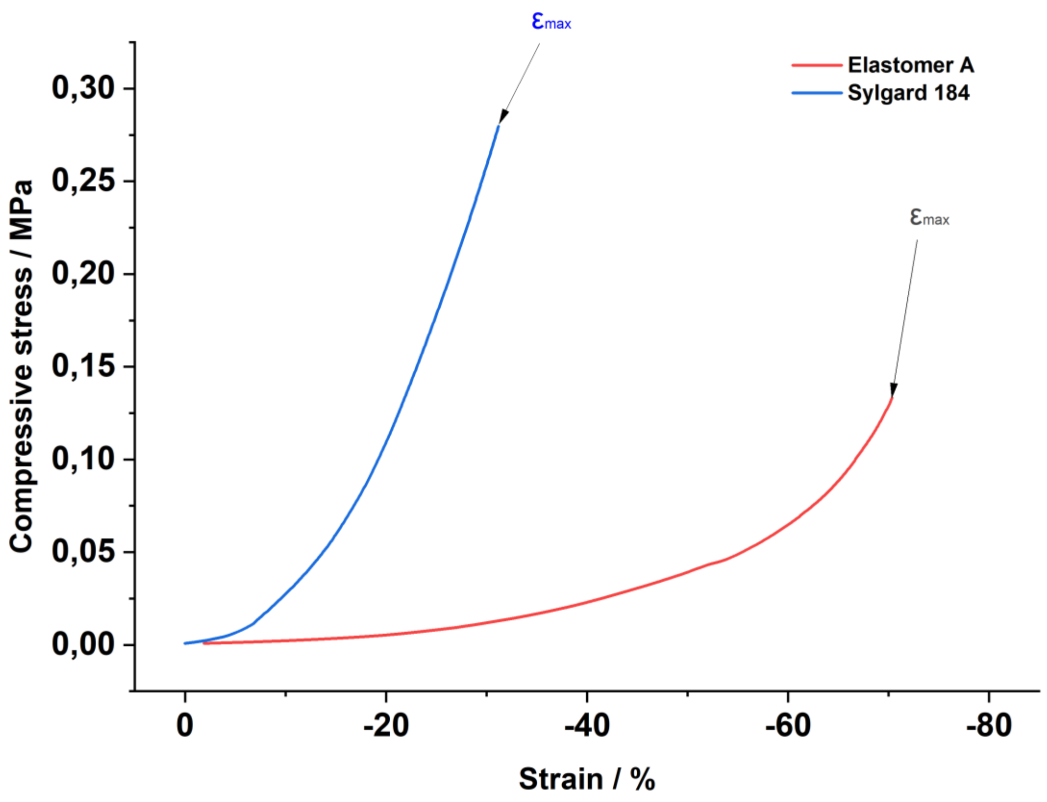

Figure SI-7. Compression tests of elastomer **A** and Sylgard 184.

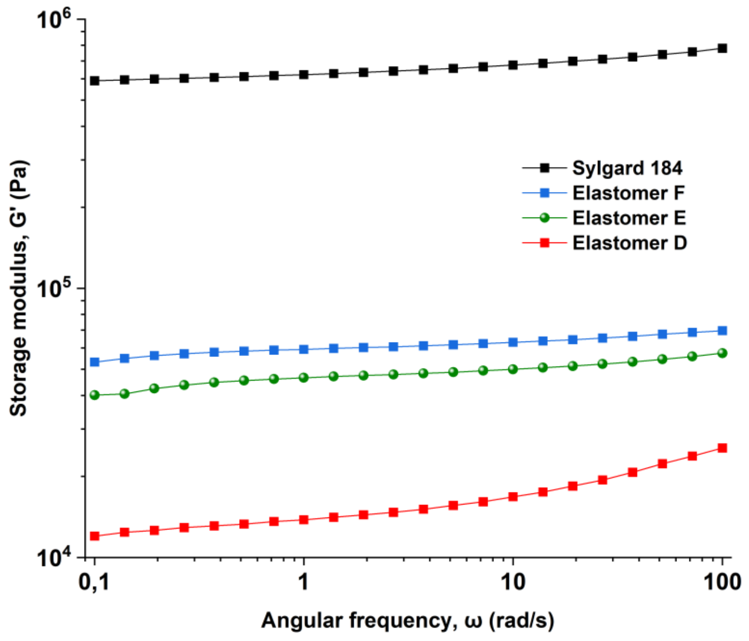

Figure SI-8. Frequency sweeps of elastomers **D-F**, cured by photoinitiated thiolene reaction, used for the ball drop experiment. Elastomer **F** without any brush content shows the highest modulus, elastomer **E** with the same grafting density as **D** but without PNF content has a significantly higher modulus.

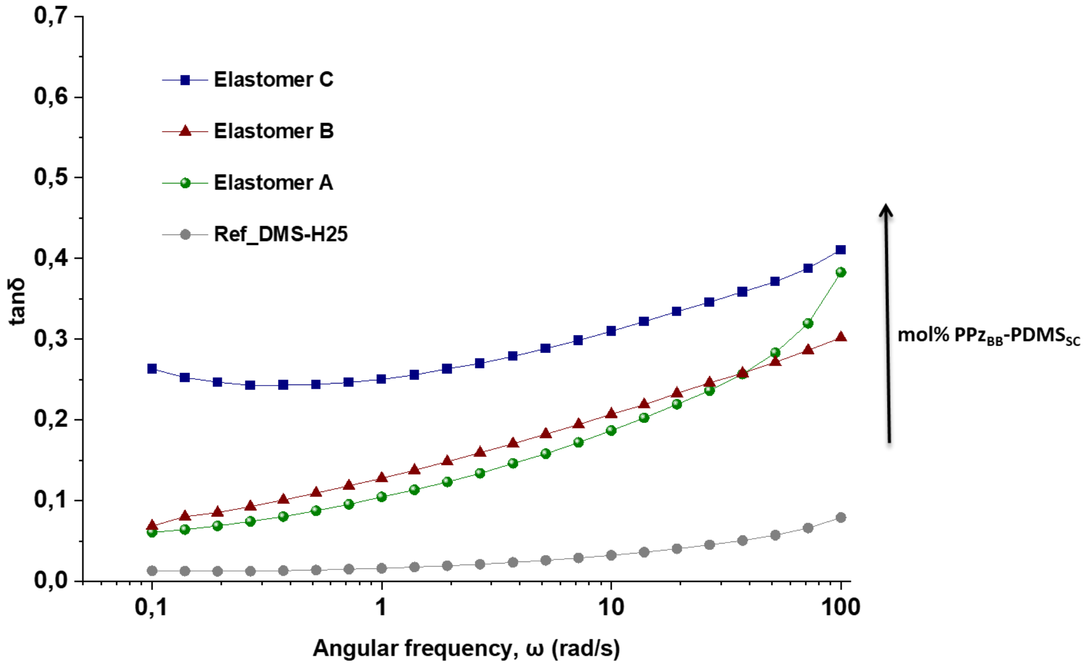

Figure SI-9. Loss factors of the platinum-catalyzed crosslinked elastomers **A**, **B** and **C** over the frequency range 0.1-100 rad/s.

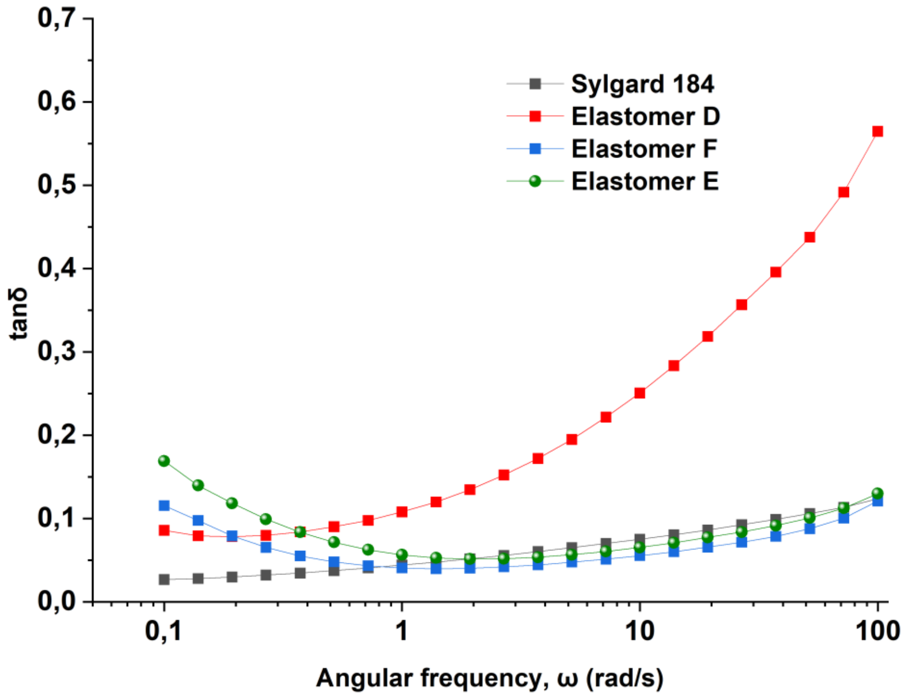

Figure SI-10. Loss factors of elastomers **D-F**, cured by photoinitiated thiolene reaction, used for the ball drop experiment.

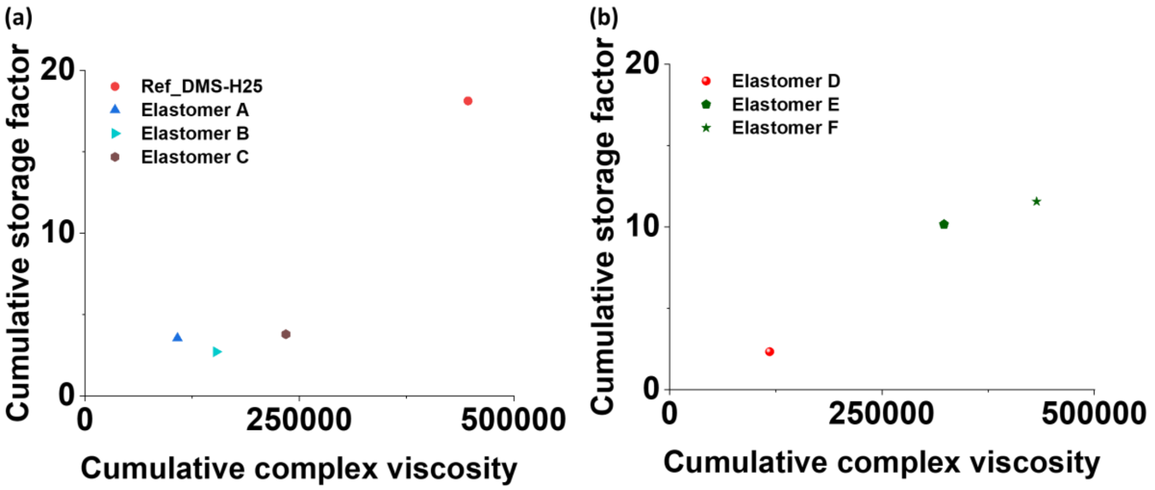

Figure SI-11. **(a)** Cumulative storage factor (CSF) of elastomers **A-C**, crosslinked by platinum-catalysis, and the reference sample Ref_DMS-H25 and **(b)** of elastomers **D-F**, cured by photoinitiated thiolene reaction.

Table SI-2. Information of functionalized PDMS chemicals used in this study.[a]

Type of chemical	Abbreviation	Mn / kDa (NMR)
**monoHydride** terminated PDMS	MCR-H21	7
**diHydride** terminated PDMS	DMS-H25	14
**monoVinyl** terminated PDMS	MCR-V21	6
**diVinyl** terminated PDMS	DMS-V21	5
**diVinyl** terminated PDMS	DMS-V31	17
**monoAminopropyl** terminated PDMS	MCR-A12	2
(**mercaptopropyl**)methylsiloxane]-dimethylsiloxane copolymer	SMS-142	4

[a]Molar mass determined by ^{1}H NMR spectroscopy.

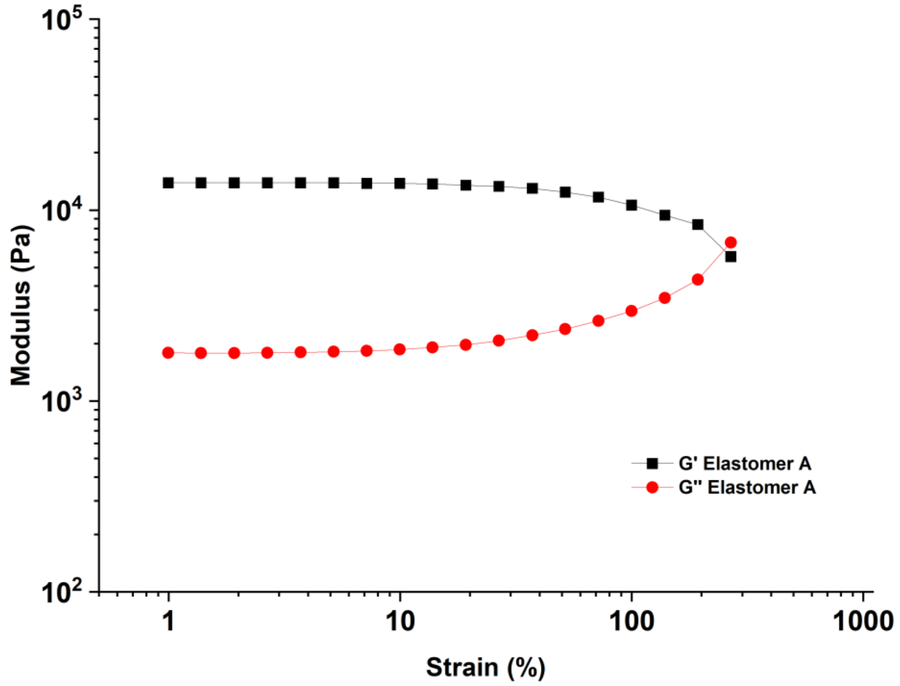

Figure SI-12. Amplitude sweep of elastomer **A** with a constant frequency of 1 rad/s and an increasing strain rate γ from 1% up to 1000% at 24°C.

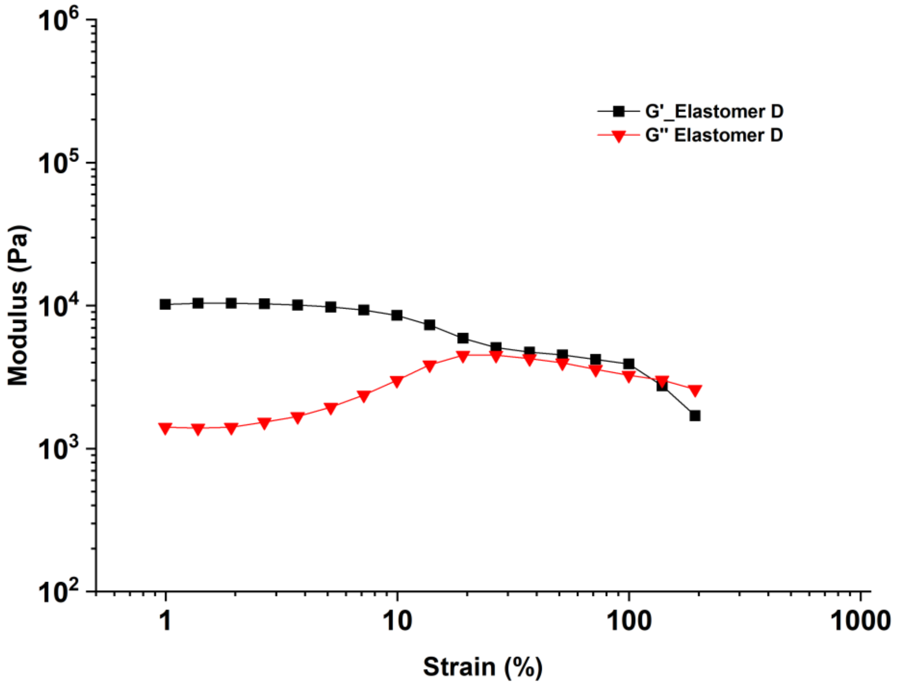

Figure SI-13. Amplitude sweep of Elastomer **D** with a constant frequency of 1 rad/s and an increasing strain rate γ from 1% up to 1000% at 24°C.

Table SI-3. Data of the ball drop experiments assessing the energy dissipation potential of the elastomers **D-F** and also Sylgard 184 as a reference. The materials were tested by dropping a steel ball (*d* = 5 mm, 0.504 g) from a height of 12.5 cm and measuring the maximum bounce height by a high-speed camera.

**Sylgard**	Rebound height / cm	dissipated Energy / J	**Elastomer F**	Rebound height / cm	dissipated Energy / J	**Elastomer E**	Rebound height / cm	dissipated Energy / J	**Elastomer D**	Rebound height / cm	dissipated Energy / J
1	5,66E-02	3,39E-04	1	5,74E-02	3,35E-04	1	4,04E-02	4,19E-04	1	1,50E-02	5,45E-04
2	5,42E-02	3,51E-04	2	5,81E-02	3,31E-04	2	3,75E-02	4,33E-04	2	1,50E-02	5,45E-04
3	5,43E-02	3,50E-04	3	5,94E-02	3,25E-04	3	3,60E-02	4,41E-04	3	1,52E-02	5,44E-04
M	5,50E-02	**3,47E-04**	M	5,83E-02	**3,30E-04**	M	3,80E-02	**4,31E-04**	M	1,50E-02	**5,45E-04**
SD	1,12E-03	5,56E-06	SD	8,37E-04	4,14E-06	SD	1,82E-03	9,04E-06	SD	9,05E-05	4,48E-07
Dissipated Energy / %	56		53			70			88		
